# Knee Kinematics Estimation Using Multi-Body Optimisation Embedding a Knee Joint Stiffness Matrix: A Feasibility Study

**DOI:** 10.1371/journal.pone.0157010

**Published:** 2016-06-17

**Authors:** Vincent Richard, Giuliano Lamberto, Tung-Wu Lu, Aurelio Cappozzo, Raphaël Dumas

**Affiliations:** 1 Univ Lyon, Université Claude Bernard Lyon 1, IFSTTAR, UMR_T9406, LBMC, F69622, Lyon, France; 2 Università degli Studi di Roma – Foro Italico, Department of Movement, Human, and Health Sciences, Rome, Italy; 3 University of Sheffield, Department of Mechanical Engineering and INSIGNEO Institute for in Silico Medicine, Sheffield, United Kingdom; 4 National Taiwan University, Institute of Biomedical Engineering, Taipei, Taiwan; 5 National Taiwan University, Department of Orthopaedic Surgery, Taipei, Taiwan; University of Zaragoza, SPAIN

## Abstract

The use of multi-body optimisation (MBO) to estimate joint kinematics from stereophotogrammetric data while compensating for soft tissue artefact is still open to debate. Presently used joint models embedded in MBO, such as mechanical linkages, constitute a considerable simplification of joint function, preventing a detailed understanding of it. The present study proposes a knee joint model where femur and tibia are represented as rigid bodies connected through an elastic element the behaviour of which is described by a single stiffness matrix. The deformation energy, computed from the stiffness matrix and joint angles and displacements, is minimised within the MBO. Implemented as a “soft” constraint using a penalty-based method, this elastic joint description challenges the strictness of “hard” constraints. In this study, estimates of knee kinematics obtained using MBO embedding four different knee joint models (i.e., no constraints, spherical joint, parallel mechanism, and elastic joint) were compared against reference kinematics measured using bi-planar fluoroscopy on two healthy subjects ascending stairs. Bland-Altman analysis and sensitivity analysis investigating the influence of variations in the stiffness matrix terms on the estimated kinematics substantiate the conclusions. The difference between the reference knee joint angles and displacements and the corresponding estimates obtained using MBO embedding the stiffness matrix showed an average bias and standard deviation for kinematics of 0.9±3.2° and 1.6±2.3 mm. These values were lower than when no joint constraints (1.1±3.8°, 2.4±4.1 mm) or a parallel mechanism (7.7±3.6°, 1.6±1.7 mm) were used and were comparable to the values obtained with a spherical joint (1.0±3.2°, 1.3±1.9 mm). The study demonstrated the feasibility of substituting an elastic joint for more classic joint constraints in MBO.

## Introduction

The *in-vivo* assessment of lower limb joint kinematics is generally performed using an optoelectronic system and skin markers. Data processing often includes a multi-body optimisation (MBO) procedure [1–10]. The principle is to minimise the sum of the squared distances between the measured and model-derived skin marker trajectories. The model-derived trajectories rely on a set of joint constraints modelling the osteoarticular structures (i.e., cartilage surfaces, capsule and ligaments). It is assumed that these joint constraints can help compensate for the soft tissue artefact (i.e., relative movement between the skin-markers and the underlying bone: STA).

STA represents the principal stumbling block in bone pose estimation [11], and its quantification and compensation demand constant effort. As a consequence of the STA, it is commonly admitted that movement analysis using optoelectronic systems and skin markers, and state-of-the art data processing displays a resolution in the order of 5 to 12° and 5 to 17 mm [11,12]. During function, human joints undergo rotations and translations the amplitude of which may be as small as a few degrees and millimetres. This is the case for those degrees of freedom that are stabilised by the passive periarticular structures and, as such, of special interest in clinical applications involving orthopaedic reconstructive procedures [13]. As a consequence, in these applications it is desirable that methods used to reconstruct these movements display resolutions in the order of 1° and 1 mm. If the objective is motor function assessment, lower resolutions may be acceptable.

Different joint constraints, which may be referred to as “hard” constraints, for use in MBO have been proposed and evaluated. The very first consisted in a spherical joint and was used to model the lower limb [6]. The knee joint has also been modelled as a hinge joint [5,9,10] and the ankle joint as a universal joint [5,9,10]. Moreover, in order to better represent the interaction between the knee osteoarticular structures, coupling curves between the joint degrees of freedom (DoFs) [1–4] and parallel mechanisms [8,14] have been proposed.

The accuracy of kinematics that can be achieved using MBO and the above-mentioned joint models is still under debate [2,7,15–17]. Barring input data errors and STA, the accuracy of the estimated joint kinematics depends on how biofidelic the joint models are. In this respect, two means of improvement are possible. One way is to model joint constraints using subject-specific information derived from medical imaging (typically bone geometry). This method has been shown to have some beneficial effect on the results [4,14,18]. However, the techniques involved, such as MRI, bi-planar fluoroscopy, or bi-planar radiography, are cumbersome. When such experimental approach is not possible, the mathematical identification of the geometrical parameters of the model is used in some advanced MBO methods [10,19]. However, this identification remains limited to the position of spherical joint centres or the orientation of hinge joint axes. A second way of improving joint kinematics estimation is to introduce “soft” constraints and a penalty-based method [17], thus accounting for ligament deformability and inter-individual differences. The use of “soft” constraints has been proposed for both the lower limb and the upper limb and provided promising results [17,20,21].

Making similar use of “soft” constraints, the objective of the present study is to introduce into the MBO another tool for modelling the osteoarticular structures: the joint stiffness matrix. Various notions of stiffness have been used in the literature and a terminology has been proposed to distinguish the different spring-like systems according to their physical nature and the method of measurement [22]. In this study, stiffness is defined as the characteristic of a 6DoFs elastic system, for which elastic forces provide resistance to the external forces, measured at equilibrium without energy dissipation. This definition of stiffness dispels confusion regarding *apparent stiffness* and *quasi-stiffness* [22]. Knee joint stiffness has been widely studied in the past [23–26], but never considered as a joint constraint for the lower limb.

This study characterises knee joint restraints by their elastic energy derived from the 6DoFs stiffness matrix. The assumption is that minimising the deformation energy of these restraints during movement is a plausible physiological criterion that may provide more accurate kinematics estimates than obtained with the normally used above-mentioned models.

The present study is based on the following hypotheses. Given the acknowledged experimental errors and STA, (a) an improvement of the accuracy of the estimated knee kinematics can be obtained by introducing joint constraints, however (b) not those constraints that impede or prescribe joint displacements, and (c) the introduction of “soft” constraints based on a joint stiffness matrix represents an acceptable biofidelic solution. The accuracies with which joint kinematics can be estimated using the joint stiffness matrix and classical joint models, as embedded in MBO, were assessed and submitted to comparative analysis. This was done using STA affected data, collected during stair-ascent using stereophotogrammetry and skin-markers, and reference data, virtually free of STA, simultaneously acquired using bi-planar fluoroscopy. Bland-Altman analysis was conducted to substantiate the above-listed claims. Furthermore, for point (c), a sensitivity analysis was performed to confirm that a single stiffness matrix can be representative of knee joint restraints, despite variability due to joint movement, muscle loading and subject-specific factors.

## Materials and Methods

### Multi-body optimisation

**Fig 1:**
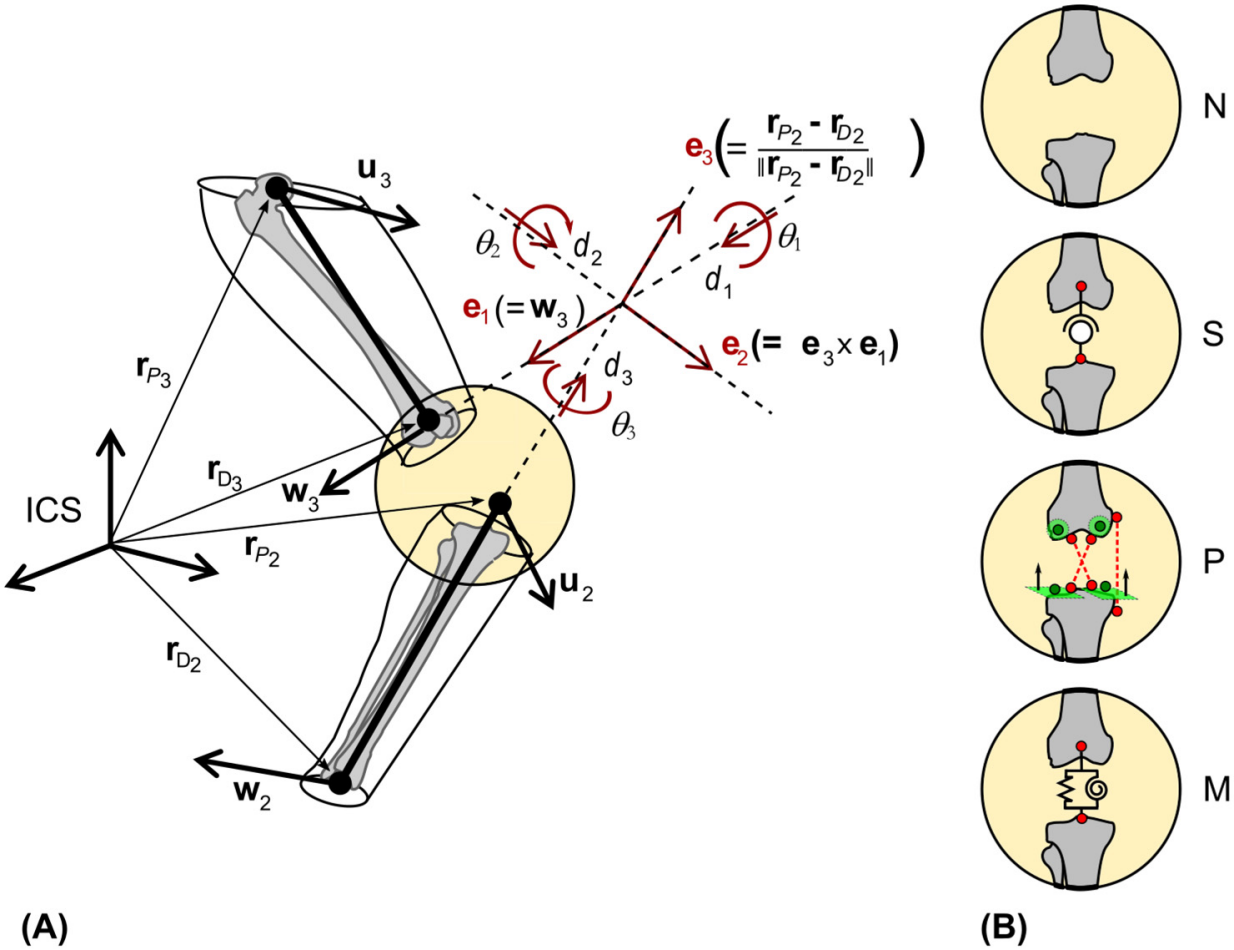
Model specifications. (A) Natural coordinates, **Q**_*i*_, for shank (*i* = 2) and thigh (*i* = 3) and knee joint coordinate system. (B) Representation of the four different knee joint models, from top to bottom: no joint model (N), spherical model (S), parallel mechanism (P), and stiffness matrix (M).

MBO is a constrained minimisation of the sum of the squared distances between measured and model-derived skin-marker positions. These distances are represented by motor constraints, **Ф**^*m*^ [8]. A nomenclature and a list of symbols used in the study are provided as supplementary material (S1 Table). The MBO was applied to two segments (thigh and shank). The design variables of the optimisation are the natural coordinates [27,28], **Q**_*i*_, consisting, for each body segment *i*, of two position vectors (proximal (*P*_*i*_) and distal (*D*_*i*_) endpoints of the segment) and two unitary direction vectors (**u**_*i*_ perpendicular to the frontal plane of the segment and **w**_*i*_ aligned with the mean flexion/extension axis of the distal joint) defining the position and orientation of segments: $Q_{i}={\left[u_{i}\;r_{P_{i}}\;r_{D_{i}}\;w_{i}\right]}^{T}$. These natural coordinates are expressed in the global (inertial) coordinate system referred to as ICS (Fig 1). Since each segment is defined by 12 parameters representing the 6DoFs, rigid body constraints **Ф**^*r*^ [28] need to be considered.

There is also the issue regarding the kinematic constraints, **Ф**^*k*^, in the different knee joint models dealt with in the present study. The first knee joint model (N) is characterised by no constraint. This represents a special case of MBO, namely a single-body optimisation (SBO) for shank and thigh separately. The MBO is defined by:

$$\begin{array}{l} \underset{Q}{\text{min}}f=\frac{1}{2}{\left[\Phi^{m}\right]}^{T}\Phi^{m}\\ \text{subject to }\Phi^{r}=0 \end{array}$$ (1)

The second knee joint model (S) is based on a spherical joint [6], and the third (P) on a parallel mechanism [8]. The MBO becomes:

$$\begin{array}{l} \underset{Q}{\text{min}}f=\frac{1}{2}{\left[\Phi^{m}\right]}^{T}\Phi^{m}\\ \text{subject to }\{\begin{array}{c} \Phi^{k}=0\\ \Phi^{r}=0 \end{array} \end{array}$$ (2)

The fourth, and original, knee joint model (M) introduced in the present study is based on the knee stiffness matrix. The MBO is modified to include a deformation energy term:

$$\begin{array}{l} \underset{Q}{\text{min}}f=\frac{1}{2}\left({\left[\Phi^{m}\right]}^{T}\Phi^{m}+w{\left[U-U_{0}\right]}^{T}S\left[U-U_{0}\right]\right)\\ \text{subject to }\Phi^{r}=0 \end{array}$$ (3)

where **U**—**U**_**0**_ is the difference between current and neutral joint angles and displacements, **S** is the stiffness matrix, and *w* is a weighting factor.

In this study, the decision to impose the same order of magnitude on both terms of the objective function resulted in arbitrarily weighting the deformation energy term by a factor *w* set to 10^−8^. The actual joint angles and displacements, **U** = [*θ*_*1*_ *θ*_*2*_ *θ*_*3*_ *d*_*1*_ *d*_*2*_ *d*_*3*_]^*T*^, are computed from the natural coordinates **Q** [29] and correspond to extension/flexion (EF), adduction/abduction (AA), and internal/external rotation (IER) angles and lateral/medial (LM), anterior/posterior (AP), and proximal/distal (PD) displacements, respectively. The goal is to conform to the joint coordinate system (JCS) definition (**e**_**1**_, **e**_**2**_, **e**_**3**_) [30]. For consistency, the same axis definitions were used for MBO, stiffness matrix determination, and processing of validation data (Fig 1).

To describe knee joint movements, tibia and femur segment coordinate systems (SCSs) were defined following the recommendations of the ISB [30], which resulted in a direct relation between the natural coordinates, **Q**_*i*_, and the SCS axes:

$$R_{0}^{i}=\left[\begin{array}{ccc} X_{i} & Y_{i} & Z_{i} \end{array}\right]=\left[\begin{array}{rrr} \begin{array}{cc} u_{i} & r_{P_{i}} \end{array} & r_{D_{i}} & w_{i} \end{array}\right]{\left[B_{i}\right]}^{-1}$$ (4)

with **B**_*i*_ being a constant transformation matrix [28].

The knee JCS was built by aligning the tibia and femur SCS axes in static position as acquired during the calibration phase. The rotation of the tibia with respect to the femur in this so-called aligned JCS was constructed as follows:

$$R_{3}^{2}={\left[R_{0}^{3}\right]}^{-1}\left[R_{0}^{2}A^{2}\right]$$ (5)

where $R_{3}^{2}$ is the rotation matrix defining the attitude of the tibia SCS with respect to the femur SCS, $R_{0}^{2}$ is the rotation matrix defining the attitude of the tibia SCS with respect to the ICS (directly related to the natural coordinates **Q**_2_) and $R_{0}^{3}$ is the rotation matrix defining the attitude of the femur SCS with respect to the ICS (directly related to the natural coordinates **Q**_3_). The alignment of both SCSs in static position is obtained by the matrix **A**^2^ consisting of the coordinates of the X, Y and Z axes of the tibia SCS in static position expressed in the femur SCS [31].

### Knee stiffness matrix

The stiffness matrix reflects the relation between the joint passive forces and moments **F** and the joint angles and displacements **U**. The stiffness matrix satisfies the general equation **F**−**F**_0_ = **S**(**U**−**U**_0_).

The present study relies on a single stiffness matrix, **S**, based on cadaveric experiments [32] conducted to target a relative orientation between femur and tibia of 45° of flexion (i.e., fourth angle condition tested on the robot, as explained hereafter). Experiments were carried out at the Institute of Biomedical Engineering in Taiwan (National Taiwan University). A fresh-frozen knee joint was obtained from a 75-year-old female (151 cm, 47 kg). Ethical approval was granted by the Institutional Research Board of China University Hospital [32]. The transepicondylar width was 73.6 mm, and the radii of lateral and medial condyles obtained by fitting two spheres on the condyles were 20 and 24 mm, respectively. The specimen, from mid-shaft femur to mid-shaft tibia, was dissected down to bone, leaving intact the major ligaments. The experimental measurements were performed using a Robot-based Joint Testing System (RJTS) consisting of an industrial robotic manipulator (RV-20A, Mitsubishi Electric Corporation, Japan) and a six-component load cell (UFS, Model PY6-100, Bertec Corporation, USA). The position and orientation repeatability of the robot were less than 0.2 mm and 0.2°, respectively. The three force and three moment components were measured thanks to the universal force-moment sensor along and about a Cartesian axis system the repeatability of which was within the range of 0.2 N for forces and 0.01 Nm for moments [32]. A dedicated computer interface, embedding a Jacobian matrix-based algorithm [33] was used to control both forces and moments, positions and orientations. Tests were carried out in seven EF angle configurations: 0, 15, 30, 45, 60, 75, and 90°. The measures were performed imposing incremental rotations and translations from neutral load equilibrium position of the knee joint, using a series of single DoF tests. Linear least-square minimisation was used in post-processing to determine, for each EF angle, the inverse of the stiffness matrix, namely the compliance matrix, while assuming a symmetric and positive matrix definition. Validation tests on the calculated matrices were carried out under a force control at 30° of EF, starting from the same neutral load position [32].

The axes of measurements in these experiments were consistent with those used for the joint angles and displacements estimated using MBO (**U** = [*θ*_*1*_ *θ*_*2*_ *θ*_*3*_ *d*_*1*_ *d*_*2*_ *d*_*3*_]^*T*^). In particular, the control of the robotic arm was operated around the JCS axes (**e**_**1**_, **e**_**2**_, **e**_**3**_) [33,34]. On the tested cadaveric knee, the femur z-axis (**e**_**1**_ in the JCS) was aligned with the transepicondylar axis and pointed toward the medial epicondyle. The y-axis was defined as the projection of the femoral longitudinal axis onto the sagittal plane pointing to the distal part of the segment. The x-axis was defined perpendicular to both the y- and z-axes. The femur and tibia SCSs were considered to be coincident in the first angle condition (i.e., 0° of flexion) tested on the robot, as explained above. The y-axis of the tibia is thus **e**_**3**_ in the JCS.

The symmetrical stiffness matrix, determined at 45° EF angle, is given by:

$$S=\left[\begin{array}{cccccc} 869.5 & 2733 & 154.2 & 55.88 & -22.45 & 81.57\\ & 8819 & 331.4 & 174.8 & -73.83 & 250.5\\ & & 129.7 & 10.36 & -1.453 & 18.48\\ & & & 3.895 & -1.620 & 5.330\\ & Sym & & & 1.246 & -1.864\\ & & & & & 8.063 \end{array}\right]\times 10^{2}$$ (6)

The neutral position should be understood as a position with minimum loads and does not imply null joint angles and displacements. The neutral joint angles and displacements, in degrees and mm, were **U**_0_ = [46.59−4.79 11.68−1.64 3.21 4.80]^*T*^. However, in order not to penalise EF in Eq 3, the neutral value was replaced by the actual joint angle, *θ*_1_. **S** was therefore reduced to the first five columns and lines of the matrix, taking no account of the coefficients relative to EF angle.

### Validation data and procedure

The validation of the study was conducted through a comparative study between the accuracies of the four MBO methods using a Bland-Altman analysis. The question whether the use of a stiffness matrix determined at a single knee flexion angle (45° of flexion) is relevant for the estimation of the stair ascent cycle was also explored through a sensitivity analysis.

#### Experimental methods

*In-vivo* stair climbing experiments were carried out at the Institute of Biomedical Engineering in Taiwan (National Taiwan University, Institute of Biomedical Engineering, Taipei, Taiwan) on two healthy male subjects who provided informed written consent to participate in the study. Approval was provided by the local Institutional Human Research Ethics Committee (please note that underlying data are not publicly available due to lack of informed consent for data-sharing at the time of collection; however, interested researchers may request data to twlu@ntu.edu.tw and obtain a de-identified, minimal dataset pending ethical approval). The age, height and mass of the subjects S1 and S2 were 21 and 20 years, 176 and 164 cm, and 84 and 59 kg, respectively. The trajectories of ten skin markers on the right thigh (four markers at mid-thigh and two on the medial and lateral epicondyles) and shank (one marker each at the head of the fibula, tibial tuberosity and medial and lateral malleoli) were recorded using a 7-camera stereophotogrammetry system (Vicon, Oxford Metrics, UK), operated at 60 samples per second. Simultaneously, bone pose was recorded with bi-planar fluoroscopy. The frequency of acquisition of the fluoroscopes (with a 1020x932 image resolution) was 30 samples per second. Stereophotogrammetric and fluoroscopic data were acquired under the same experimental conditions (same protocol, laboratory, marker set, fluoroscopy registration method, movement) as in Tsai et al. [35]. The registration method was affected by the following errors (rmse ± sd): 0.24±0.77 mm for in-plane displacements, 0.41±3.06 mm for out-of-plane displacements and 0.59±1.13° for all rotations [35].

Calibration of the reference position of the skin markers with respect to the femur and tibia was performed in a static position maintained by the subject at the beginning of the measurement session. The tibia and femur SCSs were considered to be aligned in the static position, as explained in the section *Multi-body optimisation*. The coordinate systems based on 3D bone geometry were defined in the same way as on the cadaver knee [32].

The joint angles and displacements estimated using the four MBO methods and skin-marker data were compared to their respective reference values determined using the fluoroscopy data.

#### Sensitivity analysis

This sensitivity analysis addresses the variability of the coefficients that characterise the proposed stiffness matrix using statistical distributions, and explores the propagation of this variability to the tibiofemoral kinematics estimation using the proposed MBO method. Since the stiffness matrix applied to two different subjects was computed from experimental measurements involving a single cadaveric knee at 45° of flexion, there are several potential sources of variability. First, the stiffness matrix was derived from a set of compliance matrices extracted with a range of angle conditions tested on the robot (0, 15, 30, 45, 60, 75, and 90° of flexion). However, a single stiffness matrix determined for a tibiofemoral joint presenting a flexion angle of 45° was applied to a stair climbing movement ranging from approximately 0 to 70° of flexion. Therefore, the influence of joint angle variations needs to be investigated. Second, the influence of loading conditions needs to be evaluated: the compliance matrix considered a single loading condition (almost unloaded condition), while the stair-climbing movement performed by the subjects involves varying knee loading conditions. Third, the differences between the specimen the stiffness matrix of which was used and the subjects whose movement was analysed should be taken into account. Most of these sources of variation in the stiffness matrix coefficients are not properly quantifiable, thus, to address this issue, the amplitudes of the perturbations applied to the stiffness matrix coefficients were evaluated based on the expected largest variability. A maximum ratio of 50 was found between the coefficients of the seven compliance matrices determined at each angle conditions tested on the robot [32], while the ratio between the coefficients of the knee stiffness matrix in joint loading conditions ranging from 0 to 1800 N was found to be up to 10 [36]. The sensitivity analysis was conducted to cover the largest range of variation: a range of perturbation covering 5000% of the initial value of each coefficient was applied. The stiffness matrix contains a total of 25 coefficients, but, due to symmetry, only 15 of them (5 diagonal terms and 10 extra-diagonal terms) were considered. The sensitivity analysis consisted of perturbing the above-mentioned 15 coefficients using a Gaussian statistical distribution. The means of these distributions were assumed to be the stiffness matrix coefficients determined at 45° of flexion. A Latin hypercube sampling (LHS) method was used [37] to generate a set of 1.6*10^6^ samples of perturbed coefficients. We tested for positive definiteness of the generated stiffness matrix in order to discard non-complying matrices. Finally, a run of 511 MBOs was performed, in keeping with the number of runs in previous sensitivity studies using LHS [38,39]. The Gaussian distribution of the perturbed coefficients was preserved for extra-diagonal terms of the stiffness matrix, while the distribution was truncated for diagonal terms. Indeed, these coefficients are strictly positive, resulting in the exclusion of negative terms. The actual distribution of the tested coefficients is provided in the supplementary material. For consistency of the weighting factor *w* used for the deformation energy term in the MBO, each of the perturbed stiffness matrices was scaled with respect to the initial stiffness matrix defined at 45° of flexion using the ratio of traces. The problem is formulated as follows:

$$\begin{array}{l} \underset{Q}{\text{min}}f=\frac{1}{2}\left({\left[\Phi^{m}\right]}^{T}\Phi^{m}+\left[w\cdot tr\left(S\right)\right]{\left[U-U_{0}\right]}^{T}\left[S^{*}/tr\left(S^{*}\right)\right]\left[U-U_{0}\right]\right)\\ \text{subject to }\Phi^{r}=0 \end{array}$$ (7)

where **S** is the initial stiffness matrix determined at 45° of flexion and S* is a perturbed stiffness matrix, and *tr* stands for the trace of the matrix.

#### Statistics

The hypotheses of the study were supported by Bland-Altman analysis [40,41] of the knee joint kinematics estimated using MBO embedding each of the four knee joint models (N, S, P and M). The reference kinematics, determined using fluoroscopy, assumed to be a “gold-standard” acquisition system, was set as an invariant for comparison of the four model-based methods. Given this reference condition, the difference between model-derived kinematics and fluoroscopy-based kinematics was plotted against the reference value, instead of taking the mean difference over reference and each model-derived kinematics (as initially proposed for Bland-Altman analysis) [42]. Consequently, to assess differences in the performance of the methods in the four models proposed, the Bland-Altman results for each model were compared. The method’s accuracy was given by the bias, and its precision was given by the standard deviation of the differences. Limits of agreement were set to 1.96 sd, providing an interval within which 95% of differences between model-derived and fluoroscopy-based are expected to lie. Rmse and correlation coefficients (r²) were also reported.

With regard to the sensitivity analysis, joint angles and displacements estimated through MBO for the perturbed samples of stiffness matrix were represented by the mean of the kinematics estimations over the 511 runs. Two corridors of 1 and 1.96 sd respectively around the mean value illustrate how the perturbation propagates to the kinematics.

## Results

### Comparative analysis of the knee kinematics estimates

#### Bias

**Fig 2:**
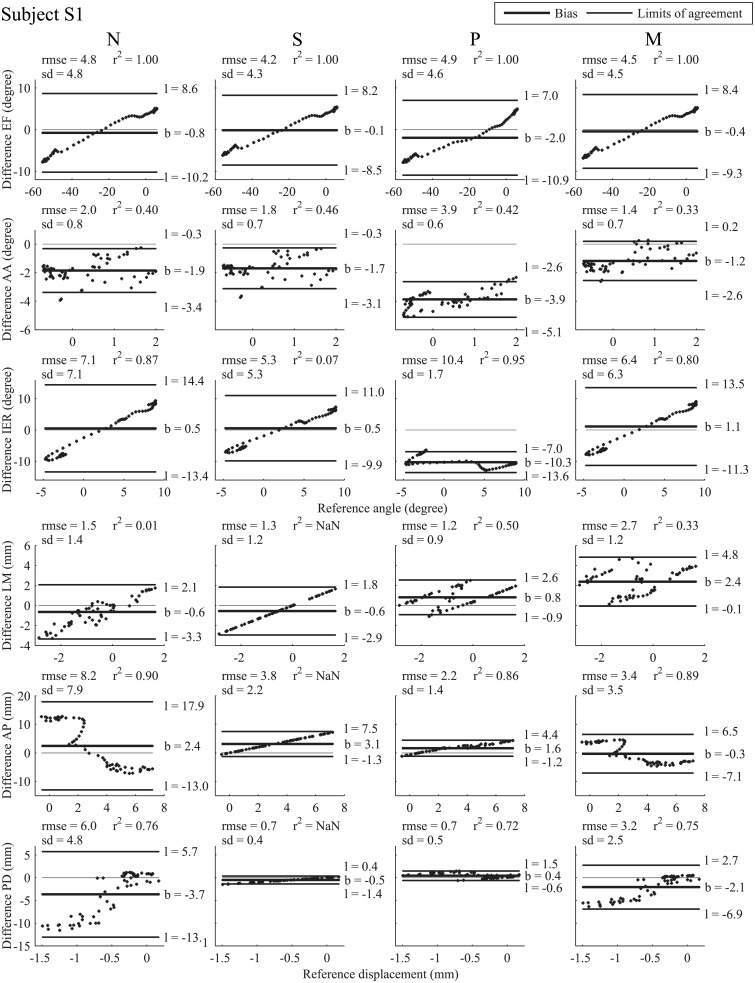
Bland-Altman plot for subject S1. Bland-Altman plot with reference kinematics (absissas) and difference (ordinates) between model-derived and reference fluoroscopy-based kinematics. From left to right Bland-Altman plots for models N, S, P, M respectively, corresponding to joint angles and displacements, from top to bottom EF, AA, IER, and LM, AP and PD, respectively. Differences between model-derived kinematics and fluoroscopy-based kinematics are plotted against reference amplitude of movement (angle or displacement). Thick black line represents the bias (mean of the differences) whose value is designated by b, thin black lines represent the limits of agreement whose value is designated by l = b ± 1.96 standard deviation. Squared Pearson’s correlation coefficient (r²), root mean square error (rmse) and standard deviation (sd) are displayed for each graph.

**Fig 3:**
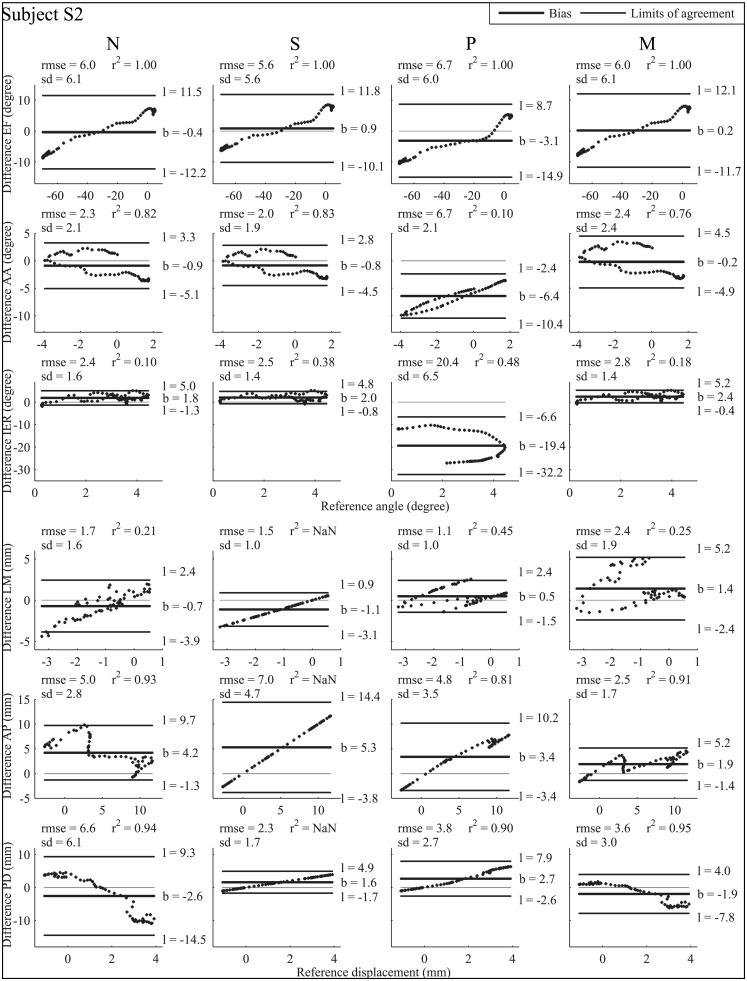
Bland-Altman plot for subject S2. Bland-Altman plot with reference kinematics (absissas) and difference (ordinates) between model-derived and reference fluoroscopy-based kinematics. From left to right Bland-Altman plots for models N, S, P, M respectively, corresponding to joint angles and displacements, from top to bottom EF, AA, IER, and LM, AP and PD, respectively. Differences between model-derived kinematics and fluoroscopy-based kinematics are plotted against reference amplitude of movement (angle or displacement). Thick black line represents the bias (mean of the differences) whose value is designated by b, thin black lines represent the limits of agreement whose value is designated by l = b ± 1.96 standard deviation. Squared Pearson’s correlation coefficient (r²), root mean square error (rmse) and standard deviation (sd) are displayed for each graph.

There was wide inter-model and inter-subject discrepancy in accuracy on knee joint angles and displacements (Figs 2 and 3 for subject S1 and S2, respectively). For EF in subject S1, the smallest bias was observed with model S (-0.1°) and the largest with model P (-2.0°), while with models N and M the bias was intermediate (-0.8 and -0.4°, respectively). In subject S2, the bias was -3.1° with model P, 0.2° with model M, and -0.4 and 0.9° with models N and S, respectively. For AA in both subjects, the smallest bias was found with model M (-1.2° for subject S1 and -0.2° for subject S2), and the largest bias was obtained with model P (-3.9 and -6.4° for subjects S1 and S2, respectively). With model N, bias was -1.9 and -0.9° for subjects S1 and S2, respectively, and with model S, bias was -1.7 and -0.8° for subject S1 and S2, respectively. For IER in subject S1, there was a bias of 0.5, 0.5, -10.3 and 1.1° with models N, S, P and M, respectively, and in subject S2 the bias was 1.8, 2.0, -19.4 and 2.4° with models N, S, P and M respectively. For LM, the bias was larger for subject S1 with model M (2.4 mm) than with models N (-0.6 mm), S (-0.6 mm) and P (0.8 mm), and larger for subject S2 with model M (1.4 mm) than with models N (-0.7 mm), S (-1.1 mm) and P (0.5 mm). For AP, the bias was smaller for subject S1 with model M (-0.3 mm) than with models N (2.4 mm), S (3.1 mm) and P (1.6 mm); the same was true for subject S2, where model M’s bias was smallest at 1.9 mm, while models N, S and P showed a bias of 4.2 mm, 5.3 mm and 3.4 mm respectively. Finally for PD, the largest bias was found with model N for subject S1 (-3.7 mm) and with model P for subject S2 (-2.7 mm), while there was a bias of -0.5, 0.4, and -2.1 mm with models S, P and M, respectively, for subject S1 and of -2.6, 1.6, and -1.9 mm with models N, S and M respectively for subject S2.

#### Standard deviation

For EF, sd values were similar with all models, being smaller for subject S1 (4.8, 4.3, 4.6, and 4.5° with models N, S, P and M, respectively) than for subject S2 (6.1, 5.6, 6.0, and 6.1° with models N, S, P and M, respectively). For AA for subject S1, sd was under 1° with all models, while for subject S2, sd was similar with models N and P (2.1°), smaller with model S (1.9°) and larger with model M (2.4°). For IER, subject S1 showed a discrepancy between sd obtained with models N (7.1°), S (5.3°), P (1.7°) and M (6.3°), while subject S2 showed less discrepancy with models N, S and M (1.6, 1.4, and 1.4° respectively) and larger sd with model P (6.5°). Standard deviations obtained for LM were 1.4, 1.2, 0.9, and 1.3 mm with models N, S, P and M, respectively for subject S1 and 1.6, 1.0, 1.0, and 1.9 mm with models N, S, P and M, respectively for subject S2. AP for subject S1 showed larger sd with model N (7.9 mm) than with models S (2.2 mm), P (1.4 mm) and M (3.5 mm), whereas for subject S2, sd was larger with model S (4.7 mm) than with models N (2.8 mm), P (3.5 mm) and M (1.7 mm). Finally, for PD, both subjects showed larger sd with model N (4.8 and 6.1 mm respectively) and M (2.5 and 3.0 mm respectively) than with models S (0.4 and 1.7 mm respectively) and P (0.5 and 2.7 mm respectively). Note that very few differences in the Bland-Altman plot were outside the 95% confidence interval.

#### Root mean square error

Similar rmses (Figs 2 and 3) were found for EF, with the four models between 4.2° (model S) and 4.9° (model P) for subject S1 and between 5.6 (model S) and 6.7° (model P) for subject S2. For AA, subject S1’s rmses were lower with models N, S and M (2.0, 1.8, and 1.4°, respectively) than with model P (3.9°). Subject S2’s rmses for AA were much higher with model P (6.7°) than with models N, S and M (2.3, 2.0, and 2.4, respectively). For IER, subject S1’s rmses were higher with model P (10.4°) than with models N (7.1°), S (5.3°) and M (6.4°). Subject S2’s rmses for IER were higher with model P (20.4°) than with models N (2.4°), S (2.5°) and M (2.8°). For LM in subject S1, the highest rmse was obtained with models M (2.7 mm), while models N, S and P had rmses of 1.5, 1.3, and 1.2 mm, respectively. In subject S2, the highest rmses were obtained with model M (2.4 mm), while rmses with models N (1.7 mm), S (1.5 mm) and P (1.1 mm) were lower. For subject S1, rmses for AP were 8.2, 3.8, 2.2, and 3.4 mm with models N, S, P and M, respectively, while for subject S2 they were 5.0, 7.0, 4.8, and 2.5 mm for models N, S, P, and M, respectively. For PD, there was wide discrepancy in results from the four knee joint models. For subject S1, rmses were between 0.7 (models S and P) and 6.0 mm (model N), with 3.2 mm for model M. For subject S2, rmses were between 2.3 (model S) and 6.6 mm (model N), with 3.6 mm for model M.

#### Correlation coefficient

The correlation coefficients were altogether high for both subjects S1 and S2 (Figs 2 and 3). For EF with all models, coefficients r^2^ were close to 1. Coefficients r^2^ were significantly lower for AA (from 0.33 for model M to 0.46 for model S) for subject S1, being much higher for subject S2 (from 0.76 for models M to 0.83 for model S), except for model P (0.10). For IER, coefficients r^2^ were generally high for subject S1 (between 0.80 for model M and 0.95 for model P), except for model S (0.07). For subject S2, coefficients r² were generally lower than for subject S1 (0.10, 0.48 and 0.18 with models N, P and M, respectively), except with model S which gave slightly higher r² (0.38). For LM, coefficient r^2^ was low, under 0.50 in both subjects S1 and S2 for models N, P and M. For AP, coefficients r^2^ were close to 0.9 for subject S1, being highest for model N (0.90) and lowest for model P (0.86). For subject S2, coefficients r^2^ were between 0.81 (model P) and 0.93 (model N). Finally, for PD, lower coefficients r^2^ were observed for subject S1 (0.76 for model N, 0.72 for model P and 0.75 for model M) than for subject S2 (0.94 for model N, 0.90 for model P, and 0.95 for model M). Note that it was not possible to compute the correlation coefficient for displacements with model S.

It should be noted that a generally satisfactory estimation of kinematics with model M is obtained around 45° of flexion, the angle at which the stiffness matrix used was obtained.

### Sensitivity

**Fig 4:**
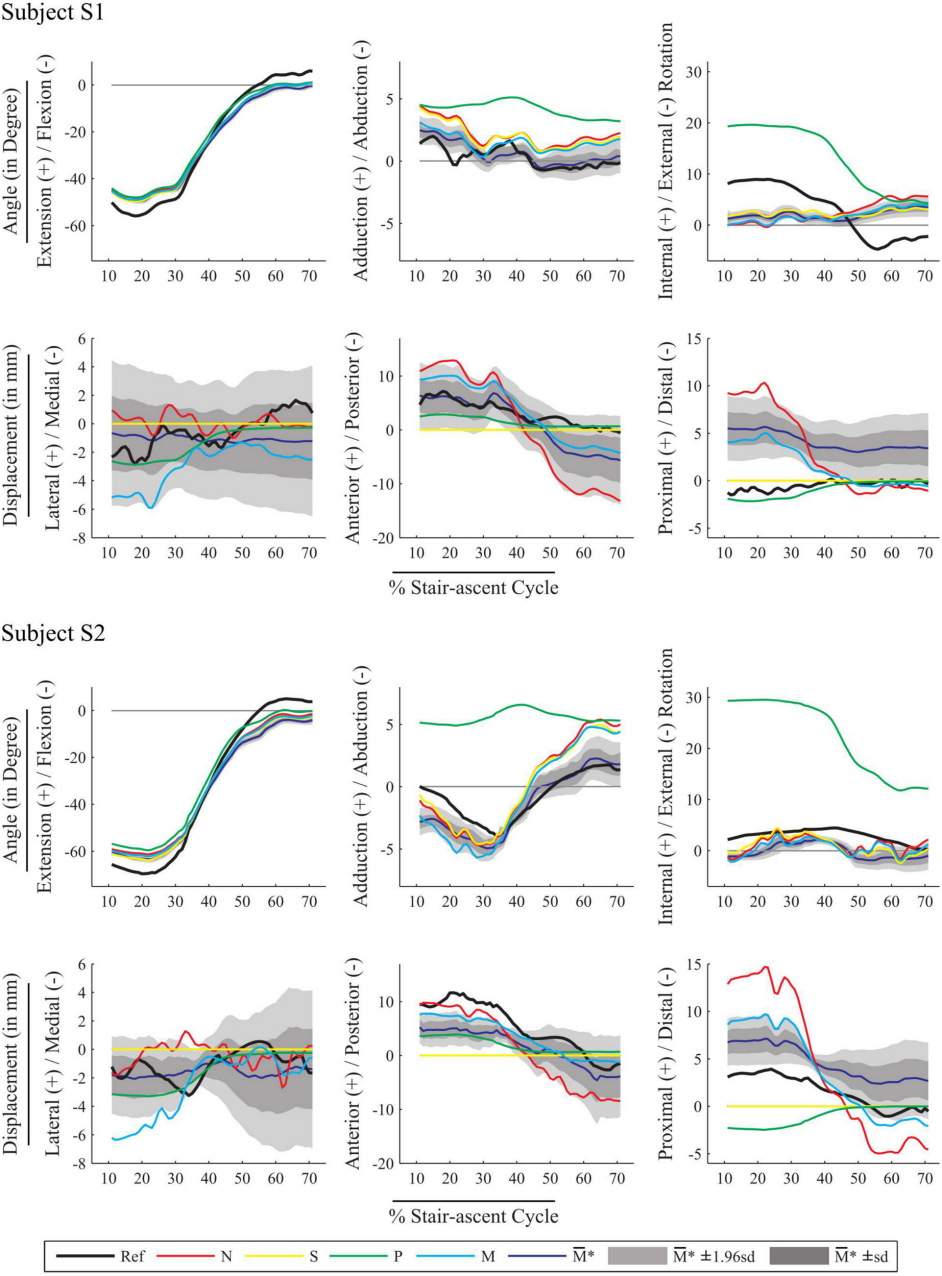
Knee joint angles and displacements, U = [*θ*_*1*_ *θ*_*2*_ *θ*_*3*_ *d*_*1*_ *d*_*2*_ *d*_*3*_]^*T*^ for both subjects. Model-derived kinematics estimated with the four knee joint models: no joint model (N, red), spherical model (S, yellow), parallel mechanism (P, green), and stiffness matrix (M, cyan) plotted against fluoroscopy-based kinematics (Ref, black). Sensitivity analysis results are represented by the mean of the kinematics estimation over the 511 runs of MBO embedding perturbed stiffness matrices ($\bar{M}*$, dark blue), with corridor representing the variation in the estimation for one standard deviation (light grey, $\bar{M}*\pm \text{sd}$) and 1.96 standard deviation ($\bar{M}*\pm 1.96\text{sd}$, dark grey) around the mean value.

The elastic joint model-derived kinematics embedding the initial stiffness matrix (M), as well as the mean ($\bar{M}*$) and corridors of 1 1 ($\bar{M}*\pm \text{sd}$) and 1.96 ($\bar{M}*\pm 1.96\text{sd}$) standard deviation of the elastic joint model-derived kinematics embedding the perturbed stiffness matrix, were computed for both subjects (Fig 4). The corridors were narrow for joint angles, with a maximum sd of 1.4° for EF and IER in subject S2, particularly for AA (0.7° for S1 and 0.9° for subject S2). Larger corridor amplitudes were observed for displacements, with a maximum sd for AP of 4.2 mm in both subjects S1 and S2. A significant difference was observed in patterns and values between the angles and displacements obtained with the initial stiffness matrix M (cyan in Fig 4) and the mean over the 511 runs on joint angles and displacements obtained with the perturbed stiffness matrices $\bar{M}*$ (dark blue in Fig 4). Curves for model M were generally within the corridor $\bar{M}*\pm 1.96\text{sd}$, except for LM and PD in subject S2.

## Discussion

Although, virtually none of the methods tested provided joint angle and displacement estimates with the desirable resolution of 1° and 1 mm, the elastic joint proved to be a feasible alternative to other joint models for embedment in MBO and improvement of the relevant outcome. The comparative analysis of the results obtained using the four selected joint models supports the hypotheses formulated in the introduction.

The first hypothesis was that an improvement of the accuracy of the estimated knee kinematics can be obtained by introducing joint constraints and performing MBO. This hypothesis is supported by the analysis of the kinematics patterns and the Bland-Altman plots as well as the results on rmse. Overall, the method with model N yields poor agreement compared to the reference, with an average of absolute bias and standard deviation of 1.1±3.8° and 2.4±4.1 mm for joint angles and displacements, respectively, with the lowest values for EF (0.6±5.5°) and LM (0.7±1.5 mm). Furthermore, rmses are systematically high, relative to the amplitude of the joint angle or displacement considered. Kinematics estimation obtained from MBO embedding model N (i.e., SBO) actually reflects most of the STA, since there are no constraints to compensate for it [43].

Our second hypothesis was that accurate knee kinematics cannot be obtained with joint constraints that impede or prescribe joint displacements. Indeed, using a spherical knee joint model, all displacements are set to zero arbitrarily, possibly resulting in better rmses but physiologically meaningless [17,18]. Moreover, in the literature, the spherical knee joint model generally results in a higher level of errors compared to the errors found in the present study [7,15,18]. Likewise, the prescription of joint angles and displacements in model P led to even greater inaccuracy in the estimation of knee joint kinematics. Indeed, statistical analysis revealed a significant bias in the kinematics obtained with this model compared to the reference (average of absolute bias and standard deviation for rotations and displacements: 7.7±3.6° and 1.6±1.7 mm), which suggests a large systematic error. This assumption is supported by the shift observed in the kinematics plot (Fig 4), in particular for IER. Although model P is considered a physiological joint model [8], it relies on “hard” constraints that prescribe two joint angles and three joint displacements, possibly resulting in high coefficients r^2^ (IER for subjects S1 and S2), but also high rmse. This frequently occurs when using models based on anatomical features which require personalisation. Substantial systematic errors associated with high correlation has also been reported for an MBO that models the knee joint using coupling curves between DoFs [2].

Our third hypothesis was that the introduction of “soft” constraints based on the joint stiffness matrix represents a promising trade-off. Introducing a stiffness matrix in MBO has previously been proposed for the spine [44,45], where no classic joint model is applicable. However, to the best of the authors’ knowledge, this approach has not been extended to other joints. Using “soft” constraints, as previously shown with deformable ligament in a parallel mechanism [17], provides mixed results. The Bland-Altman analysis (Figs 2 and 3), the rmse and the r², showed that, overall, better agreement with the reference can be obtained with model M than with model N. The rmses obtained with models M and S are similar (average level of errors for rotations and displacements: 3.9±3.6°, 3.0±2.3 mm and 3.6±3.2°, 2.8±1.9 mm, respectively), and smaller than those obtained with models N and P (average level of errors: 4.1±3.8°, 4.8±4.1 mm and 8.8±3.6°, 2.3±1.7 mm, respectively). However, the Bland-Altman analysis also indicated that the limits of agreement with respect to the reference are slightly larger with model M than with model S, while the bias is more or less equivalent. This confirms that minimising deformation energy represents at least an equally accurate alternative to the classic kinematic constraint (model S) for estimating knee joint angles such as EF and IER. The model based on the stiffness matrix also seems to estimate joint displacements efficiently (in particular for AP and PD) compared to model P. The advantage of characterising the knee joint by a stiffness matrix is the ability to define coupling between DoFs (i.e., extra-diagonal terms). The drawback lies in the introduction of a penalty-based method where the choice of the weight factor *w* is critical. Here, in order to minimise errors on both skin marker trajectories and deformation energy, we chose to consider a similar contribution to the objective function for both terms. It would be possible to consider “softer” or “harder” constraints by adjusting the weighting factor.

The sensitivity analysis revealed that the model-derived kinematics is not very sensitive to perturbation of the stiffness coefficients, which supports the use of a single knee stiffness matrix (defined at 45 degrees of flexion) throughout. Compared to the outcome of previous global sensitivity analyses performed on lower-limb multi-body models [37,38] taking into account the main parameters likely to influence performance, the stiffness matrix coefficients were shown to be less sensitive to perturbation. Model-derived kinematics have been reported to be sensitive to model parameter uncertainties, like orientation of joint axes, position of joint centres and origin, insertion and length of ligaments (in parallel mechanism) [37,38]. More detailed deformable knee models have also been found to be sensitive to ligament stiffness and reference strains [46]. In the penalty-based method proposed here, the model-derived kinematics did not depend on the absolute, but rather on the relative values of the stiffness coefficients. This made it possible for the stiffness matrix to define coupling between DoFs. However, because of the positive definite property, the ratios between coefficient values are bounded. Therefore, the substantial variation in the stiffness matrix coefficient values depending on flexion angle [32] may slightly influence the accuracy of the method, without affecting the method’s convergence, nor its feasibility. Differences between the kinematics estimated with the initial stiffness matrix (M) and the mean of the estimated kinematics over the 511 runs of MBO embedding the perturbed stiffness matrix ($\bar{M}*$) are also the consequence of the truncation of the distribution of the diagonal coefficients of the perturbed stiffness matrix.

This study is limited by the small number of subjects it considers. Moreover, the MBO method was applied to only two segments. However, as in other validation studies, reference kinematics data were available for thigh and shank only [15,17]. Further, the stiffness matrix was derived from a single cadaveric specimen. Such *in-vivo* validation data and *ex-vivo* modelling data are obviously difficult to obtain. Yet, while the inter-subject variability is not representative with only two subjects, the results of the present study confirms the feasibility of the method. Previous validation studies of MBO against fluoroscopy or pin data have been performed on two [7] to ten [2,18] subjects. Previous knee joint models, such as parallel mechanisms, have also been developed using data gathered from one cadaveric knee [17].

Moreover, the authors purposely present a single stiffness matrix to focus attention on the feasibility of such joint modelling. This choice is supported by the results of the sensitivity analysis, which show that varying the stiffness matrix coefficients does not significantly affect the performance of the method. Nevertheless, the consequence of using a single stiffness matrix obtained at 45° of flexion is a good estimation of kinematics at this specific knee position. Future implementation of bilinear stiffness (i.e., different stiffness coefficients for opposite joint movement and especially for proximal/distal displacement) or of angle-dependent stiffness, in the same way as the previously proposed angle-dependent ligament length variation [47], may lead to more accurate estimation of knee joint kinematics. Ligament-deficient stiffness matrices may also be made available, as in [32], and embedded in MBO for application to pathological subjects.

To conclude, improvement of MBO accuracy and further personalisation will necessarily follow from a better definition of joint models. In that regard, the present study allows us to foresee an alternative to the use of “hard” constraints in the multi-body method. The more physiological constraints implied by a penalty-based method (referred to as “soft” constraints) represent a progress toward making models more subject-specific.

## Supporting Information

S1 Fig Distribution of coefficients of stiffness matrix samples. Distribution of diagonal coefficients (blue) and non-diagonal coefficients (red) of the 511 samples of stiffness matrices used in the sensitivity analysis.
(PDF)

S1 Table Symbols and notation. (PDF)
