# Supplementary material for: Knee Kinematics Estimation Using Multi-Body Optimisation Embedding a Knee Joint Stiffness Matrix: A Feasibility Study
Source: PLoS One. 2016 Jun 17;11(6):e0157010. doi: 10.1371/journal.pone.0157010 (PMC4912111; doi:10.1371/journal.pone.0157010)
Supplement: S1 Fig — Distribution of diagonal coefficients (blue) and non-diagonal coefficients (red) of the 511 samples of stiffness matrices used in the sensitivity analysis. (PDF) [file pone.0157010.s001.pdf]

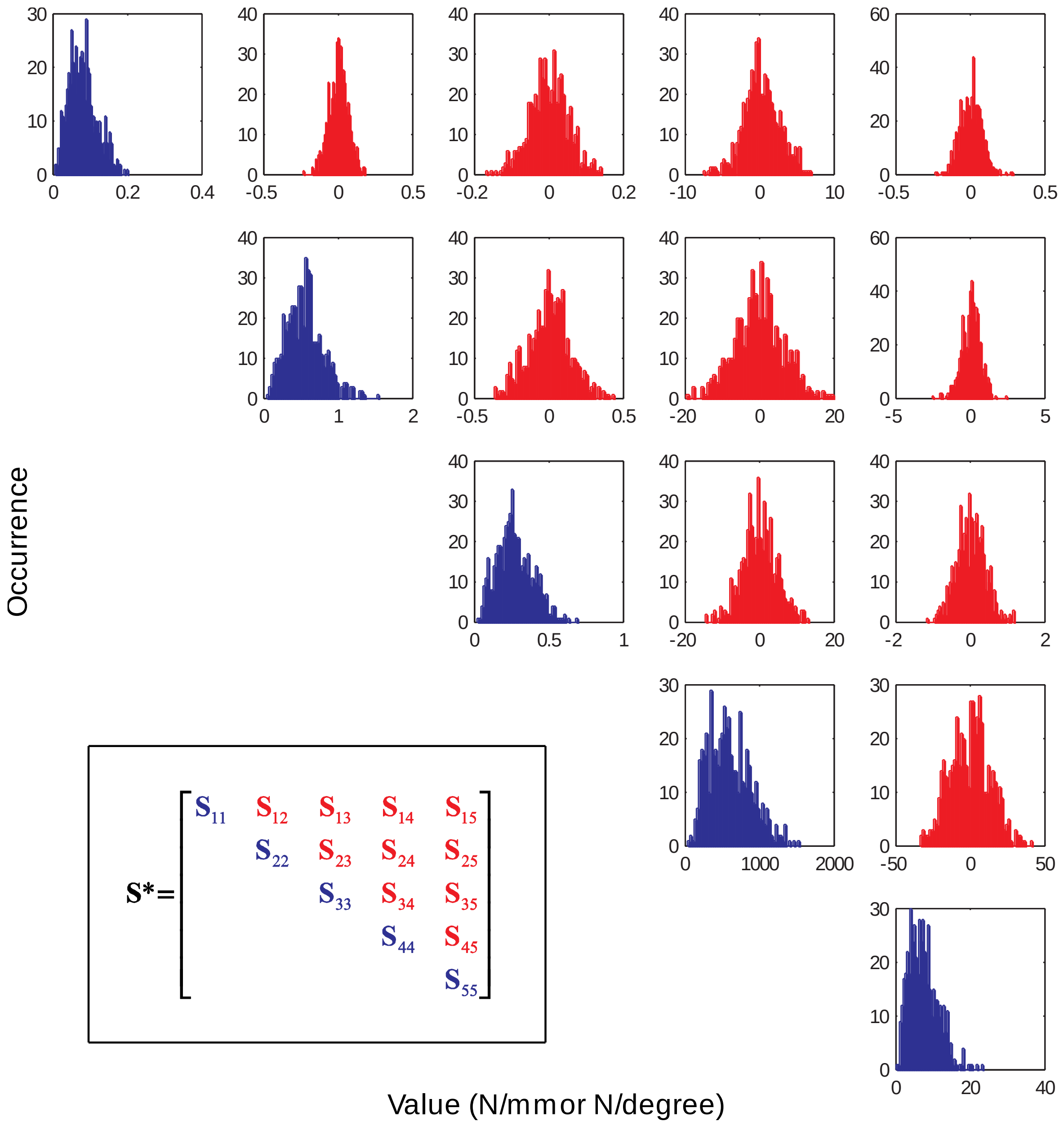

S1 Fig. Distribution of coefficients of stiffness matrix samples.

Distribution of diagonal coefficients (blue) and non-diagonal coefficients (red) of the 511 samples of stiffness matrices used in the sensitivity analysis.
