# Supplementary material for: Knee Kinematics Estimation Using Multi-Body Optimisation Embedding a Knee Joint Stiffness Matrix: A Feasibility Study
Source: PLoS One. 2016 Jun 17;11(6):e0157010. doi: 10.1371/journal.pone.0157010 (PMC4912111; doi:10.1371/journal.pone.0157010)
Supplement: S1 Table — (PDF) [file pone.0157010.s002.pdf]

Symbols	i	Body segment (i=3: shank; i=4: thigh)
	$\mathbf{Q}_i$	Generalised coordinates of segment i
	$\mathbf{B}_i$	Constant transformation matrix
	P_i	Proximal endpoint of segment i
	D_i	Distal endpoint of segment i
	$\mathbf{u}_i$	Unitary direction vector of segment *i*
	$\mathbf{r}_{P_i}$	Coordinates of the proximal endpoint of segment *i*
	$\mathbf{r}_{D_i}$	Coordinates of the distal endpoint of segment *i*
	$\mathbf{w}_i$	Unitary direction vector of segment *i*
	$\mathbf{\Phi}^m$	Motor constraints
	$\mathbf{\Phi}^k$	Kinematic constraints
	$\mathbf{\Phi}^r$	Rigid-body constraints
	f	Objective function
	$\mathbf{S}$	Stiffness matrix
	$\mathbf{U}$	Actual joint angles and displacements
	$\mathbf{U}_0$	Neutral joint angles and displacements
	$\mathbf{F}$	Actual forces and moments
	$\mathbf{F}_0$	Neutral forces and moments
	θ_1	Extension/flexion angle(degree)
	θ_2	Adduction/abduction angle (degree)
	θ_3	Internal/external rotation angle (degree)
	d_1	Lateral/medial displacement (mm)

	d_2	Anterior/posterior displacement (mm)
	d_3	Proximal/distal displacement (mm)
	$\mathbf{e}_1$	First vector of knee joint coordinate system
	$\mathbf{e}_2$	Second vector of knee joint coordinate system
	$\mathbf{e}_3$	Third vector of knee joint coordinate system
	rmse	Root mean square error
	r^2	Squared Pearson's correlation coefficient
	p	p-value
	sd	Standard deviation
	b	Bias (mean value of data)
	l	Limit of agreement (b±1.96sd)
Nomenclature	MBO	Multi-body optimisation
	SBO	Single-body optimisation
	STA	Soft tissue artefact
	DoF	Degree of freedom
	ICS	Inertial coordinate system
	JCS	Joint coordinate system
	EF	Extension/Flexion
	AA	Adduction/Abduction
	IER	Internal/External rotation
	LM	Lateral/Medial displacement
	AP	Anterior/Posterior displacement
	PD	Proximal/Distal displacement
	N	No joint model
	S	Spherical model

	P	Parallel mechanism
	M	Elastic joint model
	M*	Elastic joint model with perturbed stiffness matrix
	LHS	Latin hypercube sampling

S1 Table. Symbols and nomenclature.
